# Thorax irradiation triggers a local and systemic accumulation of immunosuppressive CD4+ FoxP3+ regulatory T cells

**DOI:** 10.1186/1748-717X-9-98

**Published:** 2014-04-25

**Authors:** Florian Wirsdörfer, Federica Cappuccini, Muska Niazman, Simone de Leve, Astrid M Westendorf, Lutz Lüdemann, Martin Stuschke, Verena Jendrossek

**Affiliations:** 1 Department of Molecular Cell Biology, Institute of Cell Biology (Cancer Research), Medical Faculty, University of Duisburg-Essen, Virchowstrasse 173, 45122, Essen, Germany; 2 Department of Infection Immunology, Institute of Microbiology, Medical Faculty, University of Duisburg-Essen, Essen, Germany; 3 Department of Radiotherapy, Medical Faculty, University of Duisburg-Essen, Essen, Germany

**Keywords:** Thorax irradiation, Regulatory T cells, T-lymphocytes, Pneumonitis, Fibrosis

## Abstract

**Background:**

Lymphocyte infiltration is a common feature of radiation-induced pneumonitis and fibrosis, but their contribution to the pathogenic processes is still unclear. Here, we addressed the impact of thorax irradiation on the T cell compartment with a focus on immunosuppressive regulatory T cells (Treg).

**Methods:**

C57BL/6 wild type mice (WT) received anesthesia only (sham controls, 0 Gy) or were exposed to a single dose of whole thorax irradiation (15 Gy). Immune cells from lung tissue, spleen, and cervical lymph nodes were collected 10 to 84 days post-irradiation and phenotypically characterized by flow cytometry.

**Results:**

Whole thorax irradiation provoked an increased influx of CD3+ T cells at 42 and 84 days post-irradiation. In contrast, local irradiation caused a sustained reduction in CD3+ T cells in peripheral lymphoid tissues. Interestingly, we observed a significant local and systemic increase in the fraction of CD4+ T cells expressing the transcription factor forkhead box P3 (FoxP3), the phenotypic marker for murine Treg, at day 21 post-irradiation. The accumulation of Treg was associated with increased levels of T cells expressing surface proteins characteristic for recruitment and immunosuppressive activity, e.g. CD103, CTLA-4 and CD73. Importantly, Treg isolated at this time point were able to suppress CD4+ effector T cells to a similar extent as Treg isolated from control mice.

**Conclusions:**

The response of the adaptive immune system to whole thorax irradiation is characterized by local immunoactivation and systemic immunosuppression. The transient accumulation of immunosuppressive CD4+ FoxP3+ Treg may be required to protect the lung against excessive inflammation-induced tissue damage. Further investigations shall define the mechanisms underlying the accumulation of Treg and their role for the pathogenesis of radiation-induced lung disease.

## Introduction

Radiotherapy is an integral part of current standard treatment concepts in oncology and provides a broad contribution to cancer cure alone and in combined treatment regimens. However, despite the high therapeutic potential of radiotherapy alone and in multimodal combinations with surgery, chemotherapy, or targeted drug therapy, a low tolerance of the normal tissue to radiotherapy can considerably limit the success of radiotherapy: Acute and late toxicity to normal tissues within the irradiated volume not only decreases the quality of life but also precludes the application of a curative radiation dose to the tumor resulting in local relapse particularly in tumors with high intrinsic radiation resistance. Hence, researchers aim to improve the therapeutic ratio by technical and physical innovations in treatment delivery, e.g. intensity-modulated radiation therapy (IMRT) or particle therapy, as well as by developing effective biology-based strategies to prevent or treat the toxic effects of ionizing radiation affecting normal tissues without increasing radiation resistance of the tumor cells.

As a clinically relevant example the lung constitutes a highly radiosensitive tissue with little repair capacity. As a consequence, radiation-induced pneumonitis and fibrosis are observed as severe dose-limiting complications of total body irradiation (TBI) or radiotherapy of thorax-associated neoplasms [1-3]. However, so far there is no available effective pharmacotherapy suited to specifically prevent or treat radiation-induced lung disease in the clinical setting so that a symptomatic anti-inflammatory therapy remains standard of care, though its use is disputed [4].

Depending on the total radiation dose and the irradiated volume, patients develop a toxic inflammation of the lung parenchyma (pneumonitis) within 4 to 12 weeks post-irradiation without or with subsequent lung fibrosis. Radiation-induced lung fibrosis is mostly observed 6 to 24 months after radiotherapy and may become chronic in patients with a large irradiated lung volume [4]. Interestingly, experimental models using whole thorax or hemithorax irradiation of fibrosis-sensitive mice (C57BL/6) mimic human disease with respect to the time course and major symptoms so that they can be used to define the underlying mechanisms as well as disease biomarkers [5-8].

Investigations in patient probes and animal models demonstrate a complex response of the lung tissue with multiple interactions between resident cells (alveolar epithelial cells I and II, endothelial cells, fibroblasts), stromal factors and infiltrating immune cells [9,10]. It is assumed that radiation-induced pulmonary fibrosis may originate from a disturbed balance between tissue inflammation and repair as it has been described for other fibrotic diseases [11]. However, it is still controversial whether cells from the innate and adaptive immune system directly contribute to radiation-induced tissue damage or only modulate disease progression.

In this regard there is evidence from preclinical and clinical investigations that T cells constitute an important part of the immune cells infiltrating the lung tissue upon irradiation of the thoracic region [6,12-15]. Even more important, the presence of CD4+ T-lymphocytes in the bronchioalveolar lavage fluid (BALF) of irradiated breast or lung cancer patients correlated with a pneumonitic reaction [13,15]. A radiation-induced increase in T-lymphocytes in the lung tissue, particularly CD4+ T-lymphocytes, during the pneumonitic phase was confirmed in rodent models [7,16,17]. Of note, depletion of CD4+ T cells during the pneumonitic phase decreased radiation-induced lung fibrosis pointing to a contribution of these cells to disease pathogenesis [16]. In contrast, lung fibrosis upon whole thorax irradiation was aggravated in *recombination-activating gene 2* (RAG2)-deficient mice; these mice lack mature T- and B-lymphocytes suggesting that lymphocytes may also have beneficial effects in radiation-induced lung disease [18]. Interestingly, in further own investigations thorax irradiation triggered the early appearance of two distinct types of T-helper cells in C57BL/6 mice, namely interleukin 17 (IL-17)-expressing CD4+ T cells and CD4+ FoxP3+ T-lymphocytes in the lung tissue [18]. The above data suggest a causal link between the recruitment or local expansion of specific T-lymphocyte populations and the course of radiation-induced lung disease. In the present investigation we addressed the potency of ionizing radiation to induce local and systemic changes in the T cell compartment with a focus on regulatory T cells (Treg) using a C57BL/6-based murine model. Treg specifically express the transcription factor FoxP3 which activates genes that silence many effector T cell genes and suppress T cell proliferation and activation in the periphery by secreting inhibitory cytokines such as transforming growth factor beta1 (TGF-β1) and IL-10 [19].

Here, we show that radiation-induced pneumonitis is associated with specific local and systemic time-dependent changes in the T cell compartment. Importantly, whole thorax irradiation (WTI) triggered the local and systemic accumulation of CD4+ FoxP3+ Treg with immunosuppressive capacities during the early pneumonitic phase. These immunosuppressive cells may be necessary to keep in check effector T cells with tissue destructive activity, such as T_H_1 cells or IL-17-expressing T_H_17 cells. An improved understanding of the underlying mechanisms and of the role of these regulatory cells during radiation-induced pneumonitis may open novel routes to prevent or treat radiation-induced pneumonitis and fibrosis.

## Material and methods

### Mouse strains

Eight-to-twelve weeks-old C57BL/6 wild-type mice (WT) were enrolled in the study. All animals were bred and housed under specific pathogen-free conditions in the Laboratory Animal Facility of the University Hospital Essen. Food consisting of a commercial laboratory animal diet and drinking water were provided *ad libitum*. The animal facility and all protocols were approved by the Universities Animal Protection Boards in conjunction with the Landesamt für Natur, Umwelt und Verbraucherschutz Nordrhein-Westfalen (LANUV) according to the German animal welfare regulations (AZ.8.87-51.04.20.09.333).

### Experimental setup for whole thorax irradiation (WTI)

For whole thorax irradiation, groups of four mice were irradiated in parallel. Animals were anesthetized with 2% isoflurane, placed in holders and irradiated simultaneously with a single dose of 0 Gy (sham control) or 15 Gray (Gy) over their whole thorax. The radiation dose was applied using a Cobalt-60 source (Phillips, Hamburg, Germany). The irradiation was performed using a field size of 23.3 cm × 2 cm at the focus target distance of 58 cm. The field size was additionally reduced to 23.3 cm × 1.5 cm field size (full width half maximum) using two Lipowitz metal absorber blocks (5.3 cm thickness) at focus surface distance of 44 cm. The beam collimation allowed for the irradiation of an axial 1.5 cm thick slice covering the lungs of 4 mice fixed in parallel position at a time. The mouse lung position within the dedicated mice holders were validated once with a CT scan of the complete positioning setup.

Dosimetry was performed with a type 31016 pin point “3D chamber” (0.016 cm^3^), a reference semiflex chamber type 31003 (0.3 cm^3^) and an electrometer type UNIDOS (PTW, Freiburg, Germany). Dose was applied with an accuracy of 3% (+3% for the two mice with 3 cm distance to central beam axis, -3% for the two mice with 9 cm distance to central beam axis). The body dose outside the irradiation field was found 1.3% of the prescribed dose. The Co-60 source provided a dose rate of approximately 0.5 Gy/min at the target.

### Collection of bronchoalveolar lavage fluid (BALF)

To obtain BAL fluid (BALF), a horizontal incision was made in the dissected tracheal tube. A syringe needle was connected and fixed by two surgical knots and the lungs were lavaged three times with 0.4 mL PBS. All fluid collected from one mouse was pooled and 50 μL were cytospun onto glass slides at 400 rpm/5 min with a Shandon Cytospin 4 (Thermo Scientific, USA). Slides were allowed to air dry for several minutes and were then Giemsa-stained. Stainings were further analysed via bright-field microscopy.

### Isolation of lymphocytes from spleen, cervical lymph nodes and lungs

Mice were sacrificed at days 10, 21, 42, or 84 post-irradiation and lung tissue, cervical lymph nodes and spleen were collected for further analysis as follows:

#### Isolation from spleen

Spleens were rinsed with an erythrocyte lysis buffer (containing 0.15 M NH_4_Cl, 10 mM KHCO_3_, and 0.5 M EDTA), meshed through a 70 μm cell strainer, passed through a 30 μm cell strainer and washed with complete medium (RPMI medium supplemented with 10% fetal calf serum, Penicilline and Streptomycine).

#### Isolation from cervical lymph nodes

Cervical lymph node cells (cLN) were disrupted with two 23G needles in PBS containing 2 mM EDTA and 2% fetal calf serum or complete medium respectively, and collected in complete medium for further analysis.

#### Isolation from lungs

Lungs were cut into pieces and digested in 1 mg/mL Collagenase D and 10 μg/mL DNAse for 45 min at 37°C and the cell suspension filtered (70 μm cell strainer) and subsequently centrifuged by 1500 rpm for 6 min. Total lung cells (TLC) were then rinsed with an erythrocyte lysis buffer (containing 0.15 M NH_4_Cl, 10 mM KHCO_3_, and 0.5 M EDTA), passed through a 30 μm cell strainer and washed with complete medium for subsequent phenotyping.

### Phenotyping of leukocytes by flow cytometry

Lung cells were stained with anti-mouse CD45 Pacific Blue (30-F11) for determination of leukocytes in the lung tissue. Splenocytes, cLN cells, and lung cells were further fluorochrome-labeled with anti-mouse CD3ϵ (145-2C11), CD4 (RM4-5), CD8 (53-6.7), CD45R/B220 (RA3-6B2), CD39 (24DMS1), CD73 (TY/11.8) and CD103 (M290). Detection of FoxP3 and CTLA-4 was performed using the FoxP3 staining kit from eBioscience (Frankfurt, Germany) with anti-mouse FoxP3 (FJK-16 s) and anti-mouse CTLA-4 (UC10-4B9), according to the manufacturer’s recommendations. All antibodies used in this study, were obtained from BD Biosciences (Heidelberg, Germany), BioLegend (Fell, Germany) or eBioscience (Frankfurt, Germany).

### RNA isolation, cDNA synthesis and RT-PCR analysis

For RNA isolation *ex vivo* isolated lung tissues were lysed in RLT-buffer using an ULTRA-TURRAX® UTC (IKA, Staufen, Germany). RNA was isolated using RNeasy Mini kit (Qiagen, Hilden, Germany) according to the manufacturer’s instruction. Total RNA (1 μg) was used for reverse transcription (RT) with Superscript™-II reverse transcriptase (Qiagen) using oligo-dT primers according to the manufacturer’s instructions. 0.5 μL of obtained cDNA was used for PCR reaction as previously described [20]. Analysis was carried out using the oligonucleotide primers FoxP3_sense CTGGCGAAGGGCTCGGTAGTCCT, FoxP3_antisense CTCCCAGAGCCCATGGCAGAAGT; βActin_sense GGCTGTATTCCCCTCCATCG; βActin_antisense CCAGTTGGTAACAATGCCATGT.

### Suppression assay

CD4+ CD25hi Treg were separated from cLNs and spleen of mice that received 0 Gy or 15 Gy whole thorax irradiation using a FACSAria II cell sorter (BD Biosciences). As responder T cells, CD4+ T cells were purified from spleens of naive WT mice using the CD4+ T cell isolation kit II (Miltenyi Biotec, Bergisch-Gladbach, Germany) and were labeled with Carboxyfluorescein succinimidyl ester (CFSE) (Invitrogen). CD4+ responder T cells (1 × 10^5^) were either cultured alone or co-cultured with CD4+ CD25hi Treg (1 × 10^5^) for 4 days in the presence of 1 μg/mL anti-CD3 (2C11; BD Biosciences). Irradiated splenocytes from naive C57BL/6 mice served as antigen-presenting cells (APCs) (3 × 10^5^).

### Statistical analysis

If not otherwise indicated, data were obtained from 2 – 3 independent experiments with at least 3 mice each. Mean values were calculated and used for analysis of standard deviation (SD) or standard error (SEM) and statistical significance. Differences were assessed by 2-way ANOVA followed by Bonferroni’s multiple comparison test. Data analysis was performed with Prism 5.0 software (GraphPad, La Jolla, CA). Statistical significance was set at the level of p < 0.05.

## Results

### WTI induces time-dependent changes in the immune cell composition of the lung tissue

**Figure 1:**
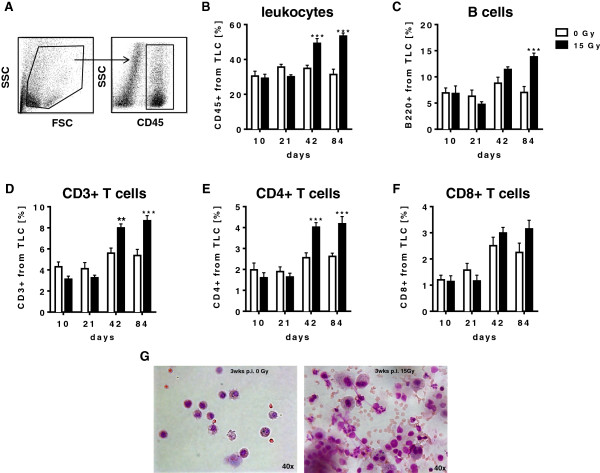
**Whole thorax irradiation (WTI) induces time-dependent local changes in the T cell compartment of the lung tissue.** C57BL/6 mice received 0 Gy or 15 Gy whole thorax irradiation. At specified time points cells were isolated from lung tissue and stained with antibodies against distinct leukocyte populations as indicated. **(A)** Gating strategy for lung cells: Living total lung cells were gated and further characterized by gating on CD45+ cells (leukocytes). All subpopulations were then gated on the CD45+ population. At different time points, cells were isolated from lung tissue, stained with antibodies against **(B)** total leukocytes (CD45+), **(C)** B-lymphocytes (B220+), **(D)** T-lymphocytes (CD3+), **(E)** CD4+ T-helper cells (CD4+) and **(F)** CD8+ T cells (CD8+) and analyzed by flow cytometry. Timelines of the indicated cell populations are shown with mean values ± SEM of percentages calculated on total lung cells (TLC). Cells of 6-9 mice per group were analyzed, ** *p* ≤ 0.01; *** *p* ≤ 0.001, two-way ANOVA followed by post-hoc Bonferroni test. **(G)** BALF (bronchoalveolar lavage fluid) was collected at 21 days post-irradiation from lungs of C57BL/6 mice irradiated with 15 Gy (right panel) and sham controls (left panel). Cytospin of BALF probes were stained with Giemsa and analyzed via bright-field microscopy: left panel: BALF from a sham control; right panel BALF from a lung irradiated with 15 Gy (40 × magnification). Pictures show one representative slide.

In a first set of experiments we compared radiation-induced local changes in immune cell composition within the lung tissue during the pneumonitic phase. To this end, we exposed C57BL/6 wild type mice to a single dose of WTI with 0 Gy or 15 Gy. Using flow cytometry we subsequently analyzed phenotypic markers of leukocytes isolated from the lung tissue 10 to 84 days post-irradiation. While the fraction of total leukocytes (CD45+ cells; gating strategy: Figure 1A) in the lung tissue was comparable to sham controls until 21 days after WTI with 15 Gy, a significant increase of CD45+ cells in the lung tissue was observed at 42 and 84 days post-irradiation (Figure 1B). The fraction of B-lymphocytes (B220+ cells) in the irradiated lung tissue also remained relatively constant during the early pneumonitic phase, but a significant increase in B220+ cells was detected at 84 days post-irradiation (Figure 1C). Finally, we also observed a pronounced increase in CD3+ T cells at 42 and 84 days post-irradiation compared to sham controls. Interestingly, the increase in CD3+ T cells was paralleled by a comparable increase in the amount of CD4+ T cells, whereas the levels of CD8+ T cells in the lungs of irradiated mice and the sham controls did not differ significantly (Figure 1D-F). Analysis of cells present in the bronchioalveolar lavage fluid (BALF) of irradiated mice and sham controls corroborated the above findings of increased immune cell infiltration into the irradiated lungs at 21 days post-irradiation (Figure 1G).

### WTI triggers distinct time-dependent changes in the T cell compartment of peripheral lymphoid organs

**Figure 2:**
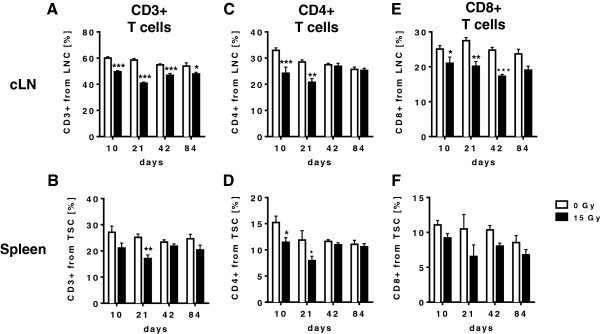
**WTI triggers distinct time-dependent changes in the T cell compartment of peripheral lymphoid organs.** C57BL/6 mice received 0 Gy or 15 Gy whole thorax irradiation. At different time points, cells were isolated from cervical lymph nodes (cLN) and spleen (TSC) and stained with antibodies against **(A/B)** CD3+ T cells (cLN and spleen), **(C/D)** CD4+ T-helper cells (cLN and spleen), **(E/F)** CD8+ T cells (cLN and spleen) as indicated. Cells were analyzed by flow cytometry and timelines of the indicated cell populations are shown with mean values ± SEM of percentages calculated on lymph node cells (LNC) or total spleen cells (TSC). Cells of 6-9 mice per group were analyzed, * *p* ≤ 0.05; ** *p* ≤ 0.01; *** *p* ≤ 0.001, two-way ANOVA followed by post-hoc Bonferroni test.

Next we examined the potential of WTI to induce systemic changes in the T cell compartment. To this end, we isolated lymphocytes from spleen and cervical lymph nodes (cLN) and analyzed the fraction of CD3+ T-lymphocytes, CD4+ T-lymphocytes and CD8+ T-lymphocytes using flow cytometry. As shown in Figure 2A and B, WTI led to a significant reduction of CD3+ T cells particularly the cervical lymph nodes (cLN) and less pronounced in the spleen of irradiated mice as compared to sham controls. The early drop in CD3+ T cells at days 10 and 21 post-irradiation involved reduction of both CD4+ and CD8+ T cells (Figure 2C-F). However, while the loss of CD4+ T cells was only transient and normal levels were reconstituted within 42 days post-irradiation, the suppressive effect of irradiation on CD8+ T cells was long-lasting and a significant reduction in the fraction of CD8+ T cells was still observed at 42 days post-irradiation, particularly in the cervical lymph nodes (Figure 2C-F).

### Thorax irradiation triggers a local and systemic accumulation of CD4+ FoxP3+ T regulatory cells

**Figure 3:**
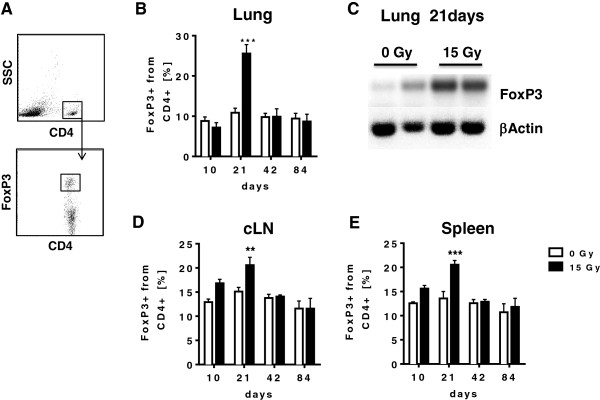
**WTI triggers a local and systemic accumulation of FoxP3+ T regulatory cells.** C57BL/6 mice received 0 Gy or 15 Gy whole thorax irradiation. At indicated time points, immune cells were isolated from lung tissue, spleen and cervical lymph nodes (cLN) and stained for flow cytometric analysis. **(A)** Gating strategy for the detection of FoxP3 on CD4+ T cells. **(B)** Treg (CD4+ FoxP3+) cells change in the lung during pneumopathy. Mean values ± SEM of the expression of FoxP3 are shown as percentages from CD4+ lung cells. **(C)** RT-PCR mRNA analysis of FoxP3 expression levels of total lung RNA isolates derived from control as well as whole thorax irradiated animals 21 days after irradiation. βActin was included as control. Two images per condition are shown. **(D/E)** Timelines of FoxP3 on gated CD4+ T cells in the cLN **(D)** and spleen **(E)** during pneumopathy. Shown are mean values ± SEM of percentages calculated on CD4+ LNC and TSC. Cells of 6-9 mice/group were analyzed; ** *p* ≤ 0.01, *** *p* ≤ 0.001, two-way ANOVA followed by post-hoc Bonferroni test.

Since our earlier investigations suggested the generation of Treg in the lungs of mice exposed to hemithorax irradiation [18], we next examined time-dependent local and systemic changes in the amount of Treg in the lung tissue and peripheral lymphoid organs of mice exposed to WTI with 15 Gy. FoxP3 is considered as a reliable phenotypic marker of Treg, at least in mice [21-23]. Therefore, we examined the fraction of CD4+ FoxP3+ T cells in lung tissue, cervical lymph nodes and spleen until 84 days post-irradiation (gating strategy Figure 3A). Interestingly, we observed a significant increase in the levels of CD4+ FoxP3+ T cells in the lungs of mice exposed to WTI compared to sham irradiated mice at 21 days post-irradiation. However this increase was only transient and levels of CD4+ FoxP3+ T cells in the lung tissue reached values of sham controls within 42 days post-irradiation (Figure 3B). The accumulation of CD4+ FoxP3+ T cells at 21 days post-irradiation could be confirmed by RT-PCR mRNA analysis of FoxP3 expression levels in total lung RNA isolated from control and whole thorax irradiated animals (Figure 3C). Interestingly, a significant accumulation of CD4+ FoxP3+ T cells at day 21 post-irradiation was also observed in the analysis of lymphocytes from cervical lymph nodes and spleen of irradiated mice. Similarly to what was observed in lung tissues this effect was only transient, though a trend to higher levels of CD4+ FoxP3+ T cells in the peripheral lymphoid organs could be already observed at 10 days post-irradiation (Figure 3D-E).

### Treg isolated from irradiated mice have normal immunosuppressive function

**Figure 4:**
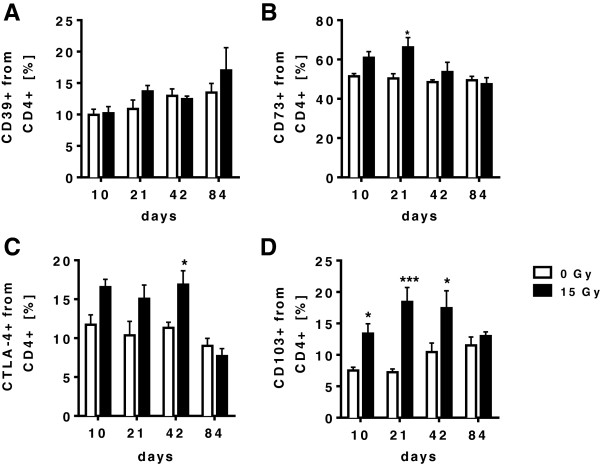
**WTI-induced changes in the T cell phenotype are associated with altered surface expression of immunoregulatory molecules on CD4+ T cells.** C57BL/6 mice received 0 Gy or 15 Gy whole thorax irradiation. At different time points, immune cells were isolated from lung tissue and stained for flow cytometric analysis. **(A)** Expression of CD39 on gated CD4+ T cells in the lung. **(B)** Expression of CD73 on gated CD4+ T cells in the lung. **(C)** Detection of CTLA-4 expression on gated CD4+ T cells in the lung. **(D)** Expression of CD103 on gated CD4+ T cells in the lung. Timelines of the indicated cell populations are shown as mean values ± SEM of percentages calculated on total lung cells. Cells of 4-6 mice per group were analyzed, * p ≤ 0.05; *** p ≤ 0.001, two-way ANOVA followed by post-hoc Bonferroni test.

**Figure 5:**
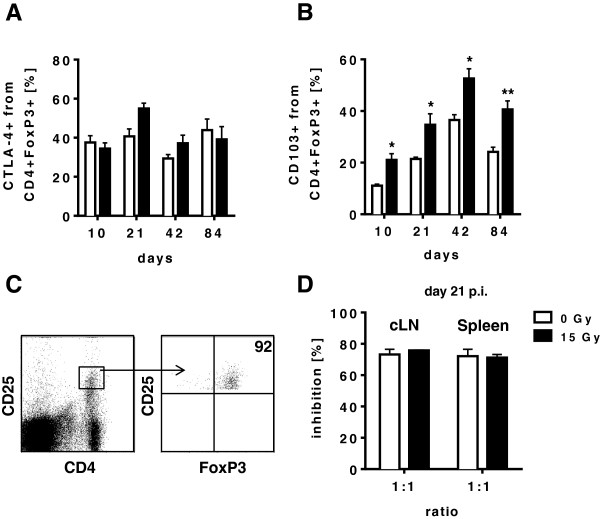
**Treg isolated from irradiated mice have normal immunosuppressive function.** C57BL/6 mice received 0 Gy or 15 Gy whole thorax irradiation. At different time points, immune cells were isolated from lung tissue and stained for flow cytometric analysis. **(A)** Detection of CTLA-4 on CD4+ FoxP3+ Treg in the lung. **(B)** Expression of CD103 on CD4+ FoxP3+ Treg in the lung. Timelines are shown as mean values ± SEM of percentages calculated on total lung cells. Cells of 4-6 mice per group were analyzed (* *p* ≤ 0.05; ** *p* ≤ 0.01; two-way ANOVA followed by post-hoc Bonferroni test). **(C)** Gating strategy for FACS-sorting of Treg from cervical lymph nodes and spleen. CD4+ CD25hi cells are 92% FoxP3+. Shown are dotplots from one representative experiment. **(D)** To determine the suppressive capability of regulatory T cells *in vitro* CD4+ CD25hi T cells (Treg) from cervical lymph nodes and spleens of 0 Gy or 15 Gy whole thorax irradiated mice were isolated at 21 days post-irradiation by FACS sorting. Treg were co-cultured at a ratio of 1:1 with CFSE-labeled CD4+ responder T cells and with antigen-presenting cells in the presence of αCD3. Proliferation of responder T cells was measured by loss of the fluorescent dye CFSE and inhibition was calculated accordingly. Data from three individual hosts are shown with mean values ± SEM.

Our data indicated that WTI leads to a local and systemic accumulation of CD4+ FoxP3+ T cells in the CD4+ T cell compartment during the early pneumonitic phase at 21 days post-irradiation. Next we aimed to explore whether CD4+ FoxP3 + cells that accumulate in the lung tissue at 21 days post-irradiation also express specific surface molecules associated with immunosuppressive activity of Treg, such as the adenosinergic ectoenzymes CD39 and CD73 [24,25]. Therefore, we performed a detailed analysis of the fraction of CD4+ T cells expressing CD39 and CD73 in the lungs of mice exposed to WTI and of sham controls. While levels of CD39+ CD4+ cells remained mostly unaffected (Figure 4A) we observed an increase in CD73+ CD4+ T cells in the lungs of mice exposed to WTI as compared to sham controls (Figure 4B). The time course of the increased surface expression of CD73 on CD4+ T cells followed the time course observed for the accumulation of CD4+ FoxP3+ T cells in the irradiated mice with a maximum at 21 days post-irradiation. Moreover, we noted a transient increase in CD4+ T cells and CD4+ FoxP3+ Treg expressing the marker proteins CTLA-4 (Figures 4C and 5A) and CD103, respectively (Figures 4D and 5B), indicative for increased recruitment and activation of these cells into the lung tissue [21,26].

Finally we explored whether Treg from irradiated mice are functional and exert suppressive activity. For this we used CD4+ CD25hi cells since sorting of viable Treg via the intracellular marker FoxP3 (fixation step) is impossible. Indeed, when comparing the phenotype and inhibitory capacity of CD4+ CD25hi cells isolated by FACS sorting from peripheral lymphoid organs of mice exposed to WTI and sham controls at 21 days post-irradiation, more than 90% of CD4+ CD25hi sorted cells expressed FoxP3 and could thus be considered as functional Treg (Figure 5C). Importantly, CD4+ CD25hi Treg isolated from cervical lymph nodes (Figure 5D left panel) or spleen (Figure 5D right panel) of mice exposed to WTI (black bars) were able to suppress proliferation of untreated CD4+ T responder cells with similar potency as CD4+ CD25hi Treg from sham controls (white bars). These results indicate that WTI triggers accumulation of Treg and that irradiation *in vivo* does not affect their immunosuppressive capacity.

## Discussion

Exposure of the thoracic region to ionizing radiation triggers time-dependent leukocyte infiltration into the lung, including lymphocytes. We show here that irradiation of the thoracic region exerts dual effects on the adaptive immune system: While WTI caused a sustained reduction in CD3+ T cells in peripheral lymphoid tissues it provoked an increased recruitment of CD4+ T cells to the lung tissue at 6 and 12 weeks post-irradiation. Importantly, we demonstrate that radiation-induced local immunoactivation was associated with local and systemic accumulation of cells with the phenotype of immunosuppressive Treg. This assumption is based on the following findings: i) WTI triggered a transient increase in the CD4+ FoxP3+ cell fraction in the lungs and the peripheral lymphoid organs of mice compared to sham controls with a maximum at 21 days post-irradiation; ii) at the time of increased FoxP3-expression the levels of CD73, CD103 and of CTLA4 on CD4+ T cells were also increased; iii) FoxP3+ expressing CD4+ CD25hi cells isolated from irradiated mice displayed enhanced expression of the marker proteins CTLA-4 and CD103 and exhibited unrestrained immunosuppressive activity.

In more detail, local irradiation of the thoracic region caused a sustained systemic suppression of CD3+ T cells in peripheral lymphoid organs that was characterized by a transient decrease in CD4+ T cells and a long-lasting reduction of CD8+ T cell numbers. These observations suggest a more pronounced sensitivity of CD8+ T cells to the cytotoxic action of IR *in vivo* compared to CD4+ T cells. Pronounced cytotoxic effects of local irradiation on the circulating lymphocyte pool had already been observed by others and had been attributed to blood flow through the radiation field [27]. In contrast, after an initial slight decrease of lymphocyte numbers in the lung tissue we observed increased levels of CD3+ T cells in the lung tissue at 42 and 84 days post-irradiation, presumably caused by an increased influx of CD4+ T cells. Thus, lymphocyte influx correlated to the time of radiation-induced pneumonitis defined by maximum impairment of lung function in our earlier investigations [6,28]. Our present data corroborate earlier findings from rodent models of thorax irradiation showing that lymphocyte numbers increase after an initial early depletion by 3 to 6 weeks post-irradiation [7,17].

It is known from preclinical and clinical investigations that CD4+ and CD8+ T-lymphocytes constitute a significant part of the immune cell infiltrate in the lung tissue of irradiated breast and lung cancer patients with a predominance of the CD4+ subset [12-16]. Of note, the increase in numbers of activated CD4+ T-lymphocytes in the BALF is more pronounced in symptomatic patients than in asymptomatic patients [13,15]. Vice-versa, increased apoptosis of peripheral blood lymphocytes, particularly CD8+ T cells, after curative radiation therapy is associated with reduced late toxicity [29]. In line with these findings, depletion of CD4+ T cells during pneumonitis decreased radiation-induced lung fibrosis in preclinical investigations in rats [16]. These findings indicate that infiltration of CD4+ T cells is a common feature of radiation-induced pneumonitis and that these cells may play a role for disease progression. Thus, CD4+ T cells may be promising targets for the modulation of radiation-induced late effects in the lung. However, so far little was known about the phenotype and function of CD4+ T-lymphocytes recruited to the lung tissue in response to thoracic irradiation. This is of particular interest because these cells depending on the microenvironment can differentiate into diverse subsets with opposing pro-inflammatory or immunosuppressive function, e.g. T_H_1, T_H_2 or T_H_17 cells and Treg, respectively.

Here we demonstrate for the first time that WTI leads to a selective accumulation of CD4+ FoxP3+ T cells both in lungs and peripheral lymphoid organs of mice at 21 days post-irradiation. These findings corroborate our recent observation about the appearance of CD4+ FoxP3+ T-lymphocytes in the lung tissue of mice exposed to hemithorax irradiation [18]. However, it had not yet been shown that local irradiation of the thoracic region also triggers a time-dependent accumulation of CD4+ FoxP3+ T-lymphocytes in cervical lymph nodes and in the spleen. The observation that local thorax irradiation also affects the T cell compartment in peripheral lymphoid organs supports earlier findings about systemic effects of a local irradiation: In this regard, irradiation of prostate tumors grown on the hint-leg of C57BL/6 mice resulted in the accumulation of CD4+ CD25hi FoxP3+ lymphocytes in peripheral lymphoid organs [30]. Moreover, others and we have shown that lymphocyte infiltration after thorax irradiation is not exclusively confined to the radiation field but can also be observed in non-irradiated parts of the lung further corroborating a systemic response of the immune system to local irradiation [6,12,13,31].

Since thorax irradiation led to a decrease in CD3+ T cells by tendency at 21 days post-irradiation, it may be speculated that the radiation-induced accumulation of Treg during the pneumonitic phase may at least partially be due to increased survival of Treg compared to T effector lymphocytes. Such enhanced resistance of CD4+ FoxP3+ cells and of CD4+ CD25hi FoxP3+ cells to ionizing radiation compared to other T-lymphocytes has recently been reported *in vitro* and *in vivo* in different experimental models [30,32-36] and had been attributed among others to enhanced expression of anti-apoptotic Bcl-2 and therefore increased resistance to apoptosis [32,33]. However, T-lymphocytes are generally characterized by a high intrinsic sensitivity to ionizing radiation so that only a minor population will survive WTI with 15 Gy. Certainly, thorax irradiation is known to provoke an increase in the levels of TGF-β1 [37], a cytokine involved in the differentiation of Treg [38-40]. Thus, radiation-induced changes in the lung microenvironment may alternatively trigger a local expansion of CD4+ FoxP3+ T-lymphocytes recruited to the lung tissue.

Of note, our data also demonstrate that Treg isolated from the lymphoid tissues of irradiated mice are fully functionally active: The CD4+ FoxP3 T cell fraction displayed up-regulated expression of surface molecules associated with recruitment and immunosuppressive function, namely CD103 and CTLA-4 [26,41,42]. In this scenario, up-regulation of CD103 on both, CD4+ T cells and CD4+ FoxP3+ suggests that Treg originate from CD4+ T cells newly recruited to the lung tissue whereas up-regulation of the adenosinergic ectoenzyme CD73 hints to immunosuppressive activity of the CD4+ FoxP3+ Treg via extracellular generation of adenosine from adenine nucleotides [24,25,43]. Our observation may provide an explanation for the suggested role of adenosine as an important mediator of tissue protection from radiation-induced injury [44,45]. Finally, the CD4+ FoxP3+ T cells isolated from irradiated mice exhibited a pronounced suppression of T effector cell proliferation that was comparable to the suppression exhibited by Treg isolated from cervical lymph nodes and spleen of sham controls.

Up to now only sparse data are available about the radiation-induced accumulation of Treg and contradictory data have been published regarding their function in the regulation of local and systemic responses to ionizing radiation. Consistent with our findings, numbers of lymphocyte subsets in peripheral blood, lymph nodes, spleens and thymuses of C57BL/6 mice decreased 2 weeks after exposure to TBI with 5 Gy, whereas the fractions of CD4+ CD25hi and CD4+ CD25hi FoxP3+ T cells in the CD4+ T cell compartment increased [32]. Though CD4+ CD25hi Treg turned out to be functional, the authors claimed a reduced immunosuppressive activity compared to Treg isolated from non-irradiated mice. Similarly, the fraction of CD4+ FoxP3+ T cells within the proliferating CD4+ T cell pool increased in response to TBI with 2 Gy but these cells displayed a reduced capacity to suppress T effector cell proliferation [33]. In line with these *in vivo* observations, human Treg isolated from the peripheral blood of healthy donors displayed a dose-dependent reduction in proliferation and immunosuppressive capacity upon *in vitro* irradiation compared to non-irradiated controls [46]. In contrast, though local irradiation of the legs of C57BL/6 mice bearing subcutaneous tumors also led to a rapid and transient increase of CD4+ FoxP3+ and CD4+ CD25hi FoxP3+ T cells in the lung and peripheral lymphoid organs, functional activity of these CD4+ FoxP3+ cells exposed to ionizing radiation *in vivo* was not affected in this experimental setting which is consistent with our present findings [30]. Altogether, these data indicate that transient local and systemic accumulation of CD4+ FoxP3+ Treg seems to constitute a common immune response to irradiation *in vivo*, though the kinetics and the functional state may depend on the radiation dose and the target tissue, respectively.

In general, Treg induced in the periphery may be considered as readout for the initiation of cytotoxic effector T cell responses known to play a key role in the maintenance of immune homeostasis and the suppression of pro-inflammatory reactions [19,47]. We therefore speculate that accumulation of Treg upon local irradiation contributes to the control of radiation-induced pneumonitis. Functional Treg may be required to keep in check effector cells of the innate and adaptive immune system, e.g. T_H_1, T_H_17 cells, thereby limiting inflammation-associated tissue damage and balancing tissue homeostasis [18,48-50].

However, when considering to target Treg for modulating the outcome of radiation-induced normal tissue toxicity it has to be taken into account that CD4+ FoxP3+ Treg may have a distinct contribution for shaping the immune response during the pneumonitic phase, which has many characteristics of acute inflammation, and the fibrotic phase characterized by chronic inflammation and tissue repair with excessive deposition of extracellular matrix and remodeling of the lung architecture, respectively [51]. In this regard, Treg dampened lung inflammation in a model of silica-induced lung disease, whereas depletion of this cell population attenuated lung fibrosis through maintenance of a T_H_1-dominated pro-inflammatory state [52]. We assume that the action of Treg in the context of radiation-induced lung disease may be similarly complex and requires further definition.

## Conclusion

Whole thorax irradiation exerts a dual effect on the adaptive immune system characterised by local immunoactivation and systemic immunosuppression. Moreover, local irradiation of the thoracic region led to a local and systemic expansion of immunosuppressive CD4+ FoxP3+ cells during the early pneumonitic phase. We speculate that these cells are needed to restrain the local cytotoxic effector T cell response induced in the lung in response to ionizing radiation thereby limiting excessive inflammation-associated lung damage and re-establishing tissue homeostasis. Further investigations shall identify the origin of these cells and the mechanisms governing their local and systemic accumulation. Moreover, the identification of the role of Treg during radiation-induced pneumonitis and fibrosis is required if we aim to exploit radiation-induced immune changes for developing effective strategies to prevent or treat radiation-induced adverse effects in the lung.

## Abbreviations

APC: Antigen presenting cell; BALF: Bronchoalveolar lavage fluid; CD39: Ectoapyrase; CD73: 5′Ectonucleotidase; CFSE: Carboxyfluorescein succinimidyl ester; cLN: Cervical lymph node; CTLA-4: Cytotoxic T-Lymphocyte Antigen 4; FoxP3: Forkhead box protein 3; Gy: Gray; IL: Interleukin; IMRT: Intensity-modulated radiation therapy; LNC: Lymph node cells; RAG2: Recombination-activating gene 2; TBI: Total body irradiation; TGF-β: Transforming growth factor beta; TLC: Total lung cells; Treg: Regulatory T cells; TSC: Total spleen cells; WT: Wild type; WTI: Whole thorax irradiation.

## Competing interest

No competing interest does exist for any of the authors.

## Authors’ contributions

VJ and FW conceived the study. FW, FC, MN and SdL participated in data collection. VJ and FW performed statistical analysis, data evaluation, and drafted the manuscript. LL helped in the experimental setup and drafting the manuscript. AW and MS critically revised the manuscript. All authors read and approved the final manuscript.
